# Enteropathy-Associated T-Cell Lymphoma Treated With Autologous Hematopoietic Stem-Cell Transplant: A Glimmer of Hope?

**DOI:** 10.14309/crj.0000000000001529

**Published:** 2024-10-10

**Authors:** Amrit Gahra, Anudeep Katrevula, Anuradha Sekaran, Balasaheb Wanve, Goutham Reddy Katukuri, Sundeep Lakhtakia, Nageshwar Reddy Duvvur

**Affiliations:** 1 Medical Gastroenterology, AIG Hospitals, Hyderabad, Telangana, India; 2 Pathology, AIG Hospitals, Hyderabad, Telangana, India; 3 Clinical Hematology, AIG Hospitals, Hyderabad, Telangana, India

**Keywords:** celiac disease, lymphoma, transplantation

## Abstract

We report a case of primary enteropathy-associated T-cell lymphoma in a 50-year-old man presenting with abdominal pain, chronic diarrhea, and significant weight loss over 6 months. Diagnosis was confirmed through endoscopy, biopsy, and positron emission tomography-computed tomography, staging the disease as stage 1E. The patient underwent initial treatment with the cyclophosphamide, doxorubicin (also known as hydroxydaunorubicin), vincristine (also known as oncovin), etoposide, and prednisolone chemotherapy regimen, followed by high-dose hyper-fractionated cyclophosphamide, vincristine, doxorubicin, and dexamethasone chemotherapy and autologous stem cell transplantation. Despite developing febrile neutropenia and septic shock during treatment, the patient achieved disease remission and symptom resolution. This case underscores the potential of autologous stem-cell transplantation as a curative approach for primary enteropathy-associated T-cell lymphoma and highlights the need for further research on its effectiveness.

## INTRODUCTION

Enteropathy-associated T-cell lymphoma (EATL) is a rare complication that affects 0.04% of patients with celiac disease (CeD). It can present with abdominal pain, diarrhea, and weight loss or, acutely, with intestinal obstruction or bowel perforation.^1^ EATL is associated with high mortality and is often diagnosed in the advanced stage, accompanied with severe malnutrition. EATL, a type of non-Hodgkin lymphoma, may complicate refractory celiac disease (RCD) type II (Secondary EATL), but it can also occur de novo in individuals with CeD (primary EATL).^2^ Here, we report a case of primary EATL successfully managed with autologous hematopoietic stem-cell transplantation.

## CASE REPORT

**Figure 1.**
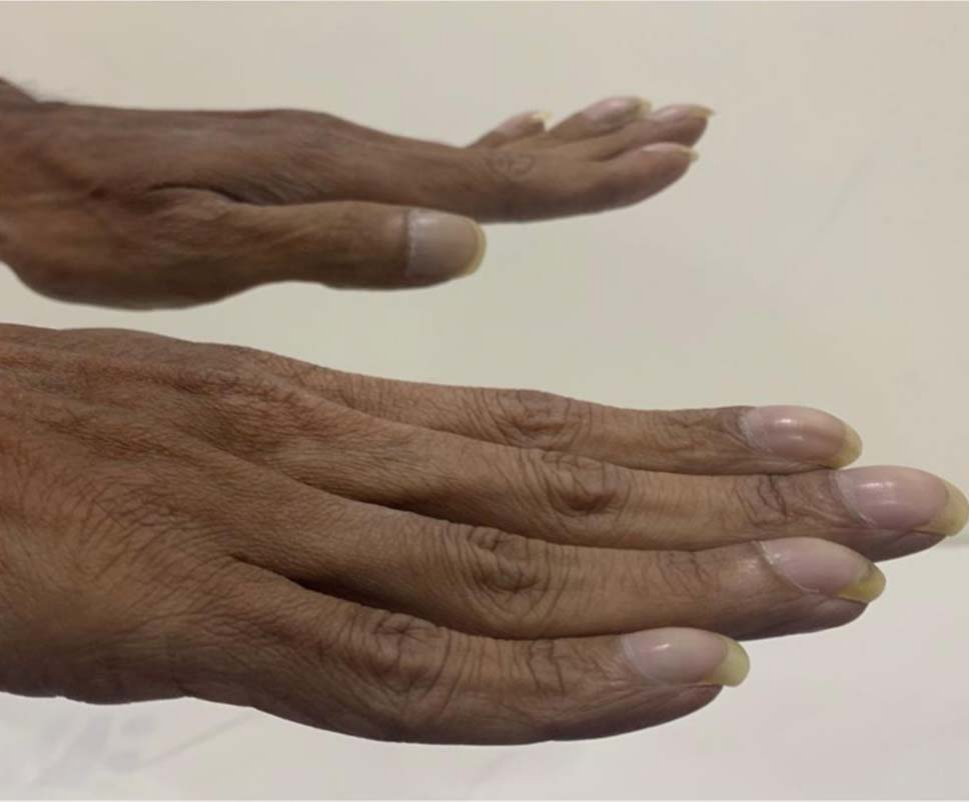
Image showing pandigital clubbing.

A 50-year-old man presented with intermittent abdominal pain, chronic watery diarrhea, and significant weight loss of 15 kg over a 6-month duration. Physical examination was unremarkable except for pallor and pandigital clubbing (Figure 1). Initial laboratory parameters showed mild iron deficiency anemia (hemoglobin: 11.0 g/dL, serum iron: 23 mg/dL, serum ferritin: 25 ng/mL, total iron binding capacity: 554 mcg/dL) and hypoalbuminemia, with a serum albumin of 2.8 g/dL. Given concerns for CeD, a serum tissue transglutaminase IgA test was performed, which was significantly elevated (157 units/mL, <20 units/mL–negative).

**Figure 2.**
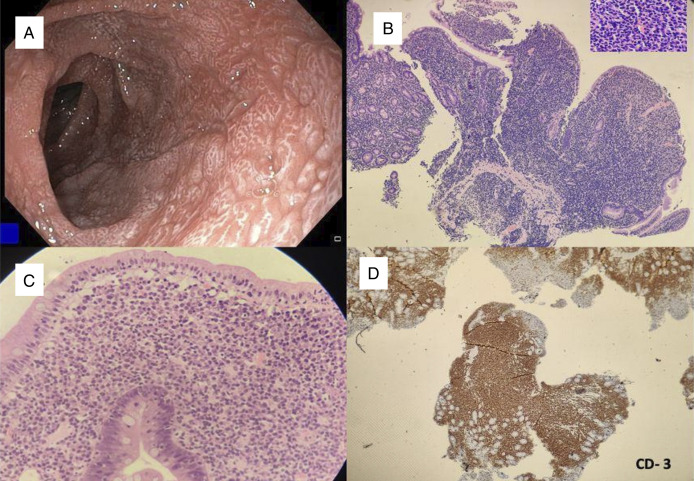
(A) Esophagogastroduodenoscopy showing loss of duodenal folds with scalloping. (B) H&E (40×) stain showing villous broadening with diffuse expansion of lamina propria by mononuclear round cells and loss of crypts. Inset: H&E (400×) showing atypical mononuclear cells. (C) H&E (100×) showing villous atrophy with intraepithelial lymphocytes. (D) Immunohistochemistry CD-3 (40×) shows diffuse positivity in lamina propria mononuclear cells. CD, cluster of differentiation; H&E, hematoxylin & eosin; PET-CT, positron emission tomography-computed tomography.

**Figure 3.**
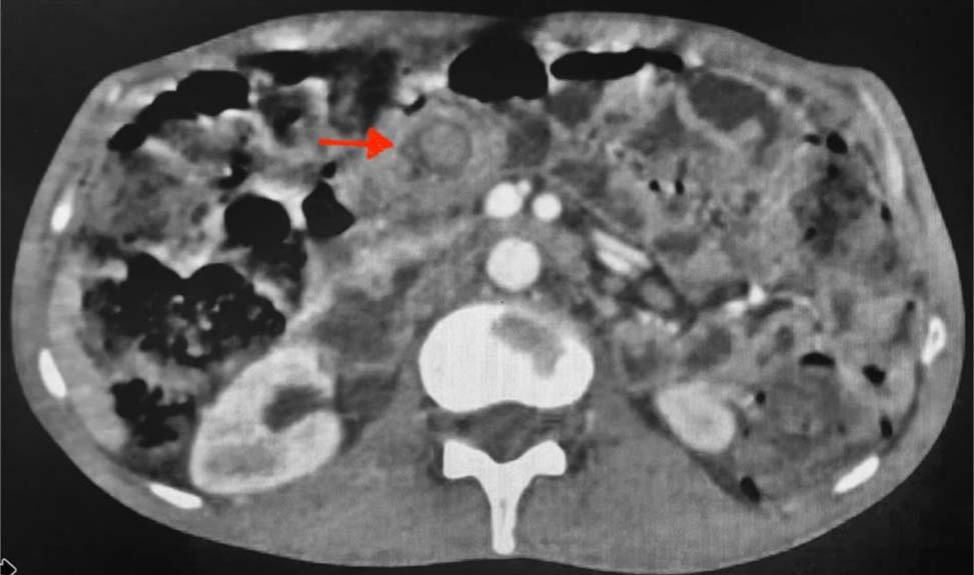
Positron emission tomography-computed tomography axial image showing heterogeneously enhancing soft tissue lesion in the proximal jejunal loop with associated intussusception (target sign).

A diagnostic esophagogastroduodenoscopy (EGD) demonstrated duodenal scalloping with thickened folds (Figure 2A). Duodenal biopsy showed villous atrophy with crypt hyperplasia and a significant increase in intraepithelial lymphocytes, along with lymphoplasmacytic cell infiltration throughout the lamina propria (Figure 2B and 2C). Immunohistochemistry revealed tumor cells positive for CD3 (pan T-cell marker) and negative for CD5, CD4, CD8, CD20, CD30, and CD56, suggesting EATL type 1 (Figure 2D). This is in correlation with morphology, confirmed EATL type-1. Positron emission tomography-computed tomography showed a low-grade fludeoxyglucose-avid soft tissue lesion (size 1.4 × 1.1 cm) in the proximal jejunal loop with associated intussusception (Figure 3). It also showed multiple tiny low-grade fludeoxyglucose-avid homogenously enhancing mesenteric nodes (maximum size 10 mm) (Stage 1E based on Lugano classification).^3^

**Figure 4.**
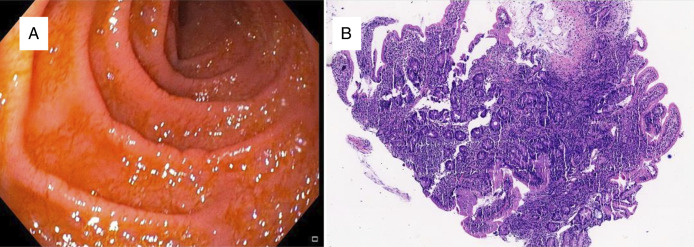
(A) (4×) esophagogastroduodenoscopy showing mild scalloping of duodenal folds. (B) H&E section shows chronic duodenitis. No evidence of lymphoid aggregates.

He was initiated on chemotherapy with a regimen of cyclophosphamide, doxorubicin (hydroxydaunorubicin), vincristine, etoposide, and prednisolone and completed 6 cycles. On repeat endoscopy, a partial morphological response was noted in mucosal disease, but the biopsy showed persistent disease. Hence, the patient underwent high-dose chemotherapy with the hyper cyclophosphamide, vincristine, doxorubicin, methotrexate, and cytarabine and dexamethasone regimen, followed by autologous hematopoietic stem-cell transplantation (ASCT). The patient developed febrile neutropenia with septic shock on day 4 of the transplant, which was managed with broad-spectrum antibiotics and supportive therapy. He gained weight with the resolution of symptoms at the 12-month follow-up. The patient was advised to continue gluten-free diet, and he reported strict adherence to it. Follow-up EGD showed mild scalloping of duodenal folds, and biopsy confirmed remission of the disease (Figure 4).

## DISCUSSION

CeD is an autoimmune condition with hypersensitivity to gluten, often presents as diarrhea. It is a global disease with a prevalence of 0.7%.^4^ While the prognosis for most individuals is generally favorable, a subset may experience significant consequences such as RCD type I and type II, ulcerative jejunitis, and EATL. EATL is a T-cell non-Hodgkin lymphoma of the intestine that originates from intraepithelial lymphocytes. Primary EATL is defined as the diagnosis of EATL in patients without a history of CeD. Secondary EATL typically presents in adult individuals who have previously been diagnosed with CeD or RCD II.^5^ Patients typically present with abdominal pain (65%–100%), diarrhea (50%–70%), weight loss (50%–70%), anorexia, vomiting, and occasionally intestinal perforation (25%–50%), obstruction, or bleeding.^6^ EATL most frequently affects the proximal jejunum and less frequently other parts of the small intestine, stomach, or colon. The diagnostic workup for EATL includes EGD with multiple duodenal biopsies, computed tomography enterography, or magnetic resonance enterography for visualizing the extraintestinal or mesenteric lymph node involvement. PET-CT scan is the imaging of choice for staging EATL using the Lugano system. The prognosis of EATL is dismal with mortality up to 85% at the end of 2 years and a 5-year survival rate ranging between 8% and 20% owing to its aggressive and chemotherapy refractory nature.^7,8^ Anthracycline-based chemotherapy obtained a 5-year overall survival rate of around 10%–20%.^9^

ASCT is the only potentially curative treatment of EATL, with an overall survival of 60% at 5 years. This is an improvement over historical outcomes.^10^ The mechanism and steps through which ASCT helps in treating EATL includes high-dose chemotherapy allowing for the administration of myeloablative doses of chemotherapy, which can overcome drug resistance and eliminate lymphoma cells more effectively than standard-dose regimens. This is followed by stem-cell rescue of previously harvested stem cells which are reinfused, allowing for rapid hematopoietic recovery, and reduction in the risk of prolonged cytopenia and associated complications. ASCT eventually causes re-setting of the immune system, potentially reducing the risk of relapse by eliminating minimal residual disease that may persist after the initial treatment. This approach allows for rapid restoration of bone marrow function by high-dose chemotherapy, overcoming drug resistance, followed by stem-cell rescue.^11^

The role of ASCT in improving outcomes for patients with EATL warrants further investigation in larger cohorts among Indian population. This case highlights the importance of early recognition and comprehensive management of primary EATL in patients with CeD. The successful use of autologous stem-cell transplantation following intensive chemotherapy demonstrates its potential as a curative strategy, offering valuable insights into treatment approaches for this challenging condition.

## DISCLOSURES

Author contributions: A. Gahra, A. Sekaran, B. Wayne, A. Katrevula performed the acquisition, analysis, or interpretation of data for the article; A. Gahra, A. Katrevula, GR Katukuri drafted the article; S. Lakhtakia, NR Duvvur made substantial contributions to the concept or design of the article and revised it critically for important intellectual content. All authors agreed to be accountable for all aspects of the work in ensuring that questions related to the accuracy or integrity of any part of the work are appropriately investigated and resolved. S. Lakhtakia is the article guarantor.

Acknowledgments: We extend our sincere appreciation to the departments of Clinical Hematology, Transfusion Medicine, and Pathology for their unwavering support and invaluable contributions to our work.

Financial disclosure: None to report.

Informed consent was obtained for this case report.
